# Effect of strip clear-cutting on the natural regeneration of *Pinus tabuliformis* plantations in northeastern China

**DOI:** 10.7717/peerj.13341

**Published:** 2022-04-28

**Authors:** Yunxia Sun, Jian Feng, Huilin Gao, Wanjin Hu, Yang Qu, Hongtao Zou, You Yin, Yuan Li, Meiyan Xin

**Affiliations:** 1 Center for Post-Doctoral Studies of Agricultural Resources and Environment, College of Land and Environment, Shenyang Agricultural University, Shenyang, Liaoning Province, China; 2 College of Forestry, Shenyang Agricultural University, Shenyang, Liaoning Province, China; 3 Liaoning Academy of Forestry Sciences, Shenyang, Liaoning Province, China; 4 Liaoning Ecological and Experimental Forest Farm, Chaoyang, Liaoning Province, China; 5 Liaoning Forestry Survey and Design Institute, Shenyang, Liaoning Province, China; 6 Baogang Greening CO., LTD, Baotou, Inner Mongolia Municipality, China

**Keywords:** Strip clear-cutting, Branch length increment, Photosynthetic capacity, *Pinus tabuliformis* plantation, Liaoning Province

## Abstract

In this study, the effect of strip clear-cutting on the natural regeneration performance of mature *Pinus tabuliformis* plantations in the three locations in western part of the Liaoning Province was analyzed. Strip clear-cutting, with clear-cut and uncut strip widths of 15, 20, 25 m, and 10 and 18 m, respectively, was conducted in spring 2014, and control, in each study location. Field investigations were conducted in 2017. Fifteen sample plots with sizes of 4 m^2^ (2 m × 2 m) were established in each clear-cut strip, uncut strip, and control. One to four saplings were randomly selected to measure the current year increment, and the lengths and numbers for branch of the first whorl. Three saplings were randomly selected from the center of the strip to measure the photosynthetic rate. Three sample plots with sizes of 4 m^2^ (2 m × 2 m) and 1 m^2^ (1 m × 1 m) were developed in each strip and control to determine the biodiversity of shrubs and herbs as well as the water content of the decomposition and semi-decomposition layer. The results show that the current year increment and branch length of the first whorl can be ordered as follows: clear-cut strips > control > uncut strips. Number of the branches of the first whorl can be ordered as follows: clear-cut strips > uncut strips > control. Strip clear-cutting was a statistically significant treatment for the current year increment and length and number of branches of the first whorl. The saplings from the clear-cut strip with a width of 25 m have the largest photosynthetic capacity compared with those from the other strips and control. The transpiration rates of the large, medium, and small saplings from clear-cut strips are the largest and those of saplings from the control are the smallest. The water content of the decomposition and semi-decomposition layer in the control is the highest, but no significant difference was confirmed between the strip clear-cutting approaches.

## Introduction

Biodiversity conservation and the improvement of natural regeneration in managed forests are important objectives in sustainable forest management (Maherali, DeLucia & Sipe, 1997; Moore & Allen, 1999; Pliūra, Kundrotas & Suchockas, 2000; Ito et al., 2006; Magalí et al., 2019). It is well known that forest harvesting methods strongly affect the overstory structure, microclimate, and soil conditions (Man & Lieffers, 1999; Flores et al., 2019). Forest harvesting approaches are also closely related to the stand regeneration, forest productivity, and biological and physical characteristics (Klenner & Sullivan, 2003; Brashears, Fajvan & Schuler, 2004; Duursma et al., 2014).

Clear-cutting and strip clear-cutting are two important forest harvesting approaches. Strip clear-cutting can be conducted by creating a small- or medium-sized opening in the canopy. Various factors, such as the tree height, canopy density, and size, shape, and orientation of the opening, influence the light distribution (Lieffers et al., 1999; Hossain & Comeau, 2019). Strip clear-cutting is the least severe anthropogenic disturbance compared to the cutting or burning for the plantation establishment (Doyon, Gagnon & Giroux, 2005; Rondon, Gorchov & Cornejo, 2008; Hidding, Tremblay & Côté, 2012; Russell, Westfall & Woodall, 2017; Kenzo, Yoneda & Ninomiya, 2018), and tends to have a rapid recovery related to destruction to forest stands or biodiversity (Faber-Landgendoen, 1992). Therefore, strip clear-cutting is effective in establishing adequate regeneration after harvesting and thus promotes sustainable forest management (Pominville & Rucl, 1995; Pothier, 2000; Hlásny et al., 2017). However, the effect of strip clear-cutting on the regeneration capacities of cut and uncut strips, especially in the zone with little precipitation is still poorly understood.

The recruitment of large saplings into the overstory is the dominant canopy recruitment process in forests; the growth of the sapling height is notably important for successful light capture (Goodburn, 2004). Architectural development at the branch level reflects the comprehensive process of tree survival and growth strategies, because the sapling architecture is a determinant of light interception and canopy photosynthesis (Rahman, Umeki & Honjo, 2014; Tanouchi & Yamamoto, 1995). Based on the response of natural saplings to different understory light levels, there is a trade-off between the ability to survive under very low light levels and rapid growth under high light levels (Williams, Messier & Kneeshaw, 1999; Mori & Takeda, 2004; Valkonen et al., 2011). The light interception of clear-cut and uncut strips differs, where the strip orientation controls the magnitude of the difference (Chen, Franklin & Spies, 1993). Therefore, quantifying the effect of strip clear-cutting with different strip widths which is related to light levels on the branch growth is important in canopy recruitment.

Photosynthesis ability difference for the natural regeneration saplings from different strip widths is an important reason leading to the growth difference (Major et al., 2009; Rubilar et al., 2013). The photosynthesis of saplings in response to canopy gap formation are closely related to the stress response to the light regime in a gap (Mulkey & Pearcy, 1992; Long, Humphries & Falkowski, 1994). To maintain a high photosynthetic activity, saplings must adapt to the various understory light interception caused by forest harvesting approaches (Mitamura, Yamamura & Nakano, 2008). In addition, the microclimate which is closely related to the strip width can also affect the genetic diversity of the generation (Pliūra, Kundrotas & Suchockas, 2000). The difference of the genetic composition of generation from large or narrow clear-cutting areas could also have further implication on the adaption of stand in the future (Baker et al., 2014). However, the analysis on the effect of strip clear-cut on photosynthesis ability of saplings and the understory biological diversity, especially for the semiarid region is limited.

The western region of the Liaoning Province is a typical zone with little precipitation and maximized water conservation and soil functions in forests. Therefore, the improvement of the natural regeneration ability of forests in this area is important. *Pinus tabuliformis* performs well with respect to water and soil conservation and environmental protection (Wang & Liu, 2011). *The Pinus tabuliformis* plantations in the Liaoning Province in northeastern China cover an area of ~0.14 million hectares. Mature *Pinus tabuliformis* plantations account for approximate 65% of the total area covered by this tree species (Gao et al., 2020). Following the study of Gao et al. (2020), the aims of this study were to (1) compare the effects of clear-cut and uncut strips on the sapling height growth for the current year, length and number of branches for the first whorl, (2) compare the effects of clear-cut and uncut strips on the photosynthetic capacities of the leaves of saplings; and (3) evaluate the effects of clear-cut strips on the biological diversity and water content of decomposition and semi-decomposition layer. The results of the present study provide references for decision-making regarding forest management strategies.

## Materials and Methods

### Study area

This research was conducted in the Liaoning ecological and experimental farm located in the semiarid area of the western part of the Liaoning Province in northeastern China (120°15–121°18E, 41°23–42°17N). Continental monsoon climate of the northern temperate zone is the typical climate of this area. The mean annual temperature is 8.3 °C and the mean annual precipitation ranges from 450 to 550 mm. The elevation ranges from 200 to 1,074 m and 145–150 days are frost-free. Brown forest soil is the main soil type in this area. *Pinus tabuliformis*, *Populus alba*, *Salix matsudana*, *Ulmus macrocarpa*, *Quercus mongolica*, and *Tilia mandshurica* are the main tree species for this region of China.

**Figure 1:**
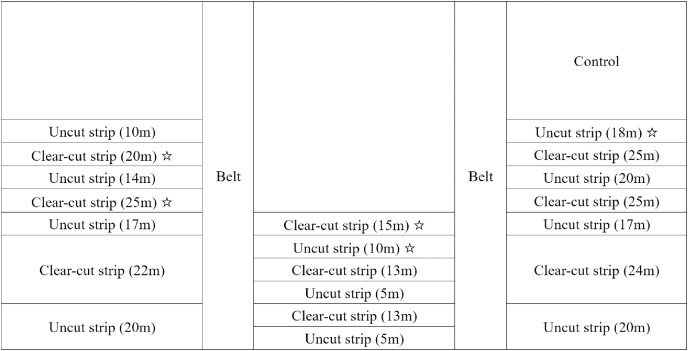
Partial scheme of the strip clear-cut experiment design in the *Pinus tabuliformis* stand in the western part of Liaoning Province. The star (☆) indicates the strips where sample saplings were obtained.

The study area is located in *Pinus tabuliformis* plantation in the Linghe experimental region (location A) from Liaoning ecological and experimental farm, Songzhangzi experimental region (location B) from Qitian forest farm, and Shahai forest farm (location C). Location B and C are within Jianping county with the distance of approximate 37 km, and Location A is from Beipiao county with the distance to Jianping of 140 km. The forest age and density for location A, B, and C are 45, 43, and 46 years and 1,035, 1,058, and 1,002 trees per hectare, respectively. In the spring of 2014, a clear-cut strip experiment was established on around 50 hectares in a mature *Pinus tabuliformis* plantation. All the three experimental locations show identical topographic characteristics with the slope smaller than 5° and southeast-facing aspect. The length of strips varied from 200 to 300 m. The orientation of the strips for the three locations were in the south–north direction. The widths of the clear-cut strips and uncut strips varied from 13 to 25 m, and 5 to 20 m, respectively. To avoid the effect of width on the results between the three locations, our investigation was conducted at the clear-cut strips with the widths of 15, 20, and 25 m and the uncut strips with the widths of 10 and 18 m for all the three locations (Fig. 1). The average mature pine height in locations A, B and C was 15.9, 15.4, and 15.5 m, respectively, and all understory trees were naturally regenerated. Harvesting was conducted as carefully as possible to avoid damage to saplings and the ground surface. Timber and slash were removed immediately after harvesting was completed.

### Data collection

Field investigations were conducted in the strip clear-cut experimental zone of *the Pinus tabuliformis* plantation in August 2017 for location A, in December 2017 for location B and C. All measurements were conducted in clear-cut strips with widths of 15, 20, and 25 m, uncut strips with widths of 10 and 18 m, as well as untreated sites (approximate 100 m × 100 m for location A, 95 m × 95 m for location B, 110 m × 100 m for location C) served as a control. In each study location, 12 to 15 sample plots were established within each treatment in the clear-cut strip, uncut strip, and control. Plots measured 4 m^2^ (2 m × 2 m), and to avoid edge effects between the clear-cut and uncut strips, all sample plots were established in the center of each strip at distances of 10 m. For each sample plot, the number of seedlings and saplings were recorded in the field. The sapling age was estimated by the number of whorls for the live branches, and the scar remaining on a stem after the branch falls along the sapling bole. Seedling was defined as a height below 30 cm and sapling was defined as a height above 30 cm and below 1.3 m. A total of 1 to 4 saplings with healthy crowns and no apparent damage to the branches was randomly selected from each sample plot. The sapling selection criterion was a distance larger than 1 m between two saplings. For each selected sapling the total sapling height and height of the leader (herein referred to as current year increment) was recorded using a measuring tape to the nearest cm. The lengths of the first whorl branches were also measured and the number of branches for the first whorl was also recorded. From each strip, one large (with the average sapling height for the 10 largest saplings), one medium (with average sapling height), and one small (with the sapling height for the 10 smallest saplings) sampled sapling in the center of the strip were randomly selected to measure the photosynthetic rate using a portable photosynthesis system (LI-6400XT; LI-COR, Inc., Lincoln, NE, USA). Two cloud-free days in August were chosen to conduct the photosynthesis measurements. The photosynthetic rates of all saplings were measured between 9:30–10:30, 11:00–12:00, 13:00–14:00, and 15:00–16:00. Measurements were conducted twice in each time period. Five continuous measurements were carried out during each measurement and their mean values were used for the analysis. To account for shrubs and herbs in each treatment, three 4 m^2^ (2 m × 2 m) sample plots were established to tally individuals at the species level. In addition, a 1 m^2^ (1 m × 1 m) plot was established to estimate the percent cover of the ground area occupied by shrubs and herbs. In all the plots we evaluated the water content of decomposition and semi-decomposition. The decomposition layer was defined as having an intact profile of leaves, while the semi-decomposition layer had no intact profile if leaves and organic material was broken down and brown. In each layer around 0.5 kg fresh weight samples were collected and oven dried at the lab at a temperature of 85 °C for 24 h to reach a constant weight and the dry weight was measured.

### Data analysis

The current year increment, branch length and number of branches of the first whorl for all sampled saplings were used to reflect the effect of clear-cut strip on the saplings growth status. The net photosynthesis rates, stomatal conductance, and transpiration rates of the leaves of each sapling were used to determine the photosynthetic characteristics of the saplings. The biological diversity and water content of the shrubs and herbs were used to characterize these understory plant communities. The biological diversity of the shrubs and herbs of each sample plot was calculated using the Shannon–Wiener index and Eq. (1).

(1) $${H_i} = - \sum\limits_{j = 1}^{N{S_i}} {({P_{ij}}\ln ({P_{ij}}))}$$

where H_*i*_ is Shannon–Wiener index, *i* = 1 is for the shrubs, and *i* = 2 is for herbs. NS_*i*_ is the number of the species for shrubs and herbs. P_*ij*_ = N_*ij*_/N_*i*_, N_*ij*_ is the number of the individuals of the *j*th species for shrubs and herbs in each sample plot, and N_*i*_ is the total number of individuals of all species for shrubs and herbs in each sample plot.

For all the three locations, one-way ANOVAs were performed to determine the influence of strip clear-cutting (15, 20, 25 m widths of clear-cut strip, 10, 18 m widths of uncut strip) and control on the growth status of saplings and the regeneration of shrubs and herbs. The effect of strip clear-cutting and time periods (9:30–10:30, 11:00–12:00, 13:00–14:00, and 15:00–16:00) on the net photosynthesis rates, stomatal conductance, and transpiration rates were analyzed with two-way ANOVAs. All analyses were performed using R software version 4.0.5 (R Core Team, 2021). HSD-Tukey was used to conduct the multiple comparisons.

## Results

### Growth status of saplings

There was almost no seedling in the plots and the average count of saplings from each sapling were 11.2, 10.9, and 11.0 for location A, B and C, respectively. The density of saplings from the clear-cut strip was the largest, and the control was the smallest for location A, B and C (Table 1). The current year increment, branch length and number of branches of the first whorl of the sample saplings from clear-cut strips, uncut strips, and control were compared (Table 2). Normality and homogeneity of variances of the data were tested to meet the requirement of ANOVAs and HSD-Tukey. On the whole, current year increment shows the following trend: clear-cut strip > control > uncut strip for location A and B, and clear-cut strip > uncut strip > control for location C. Statistically significant differences were found for the current year increment of sapling height among the different widths of the strips and the control for all the three locations (F = 5.21, *P* < 0.05 for location A; F = 4.21, *P* < 0.05 for location B; F = 2.88, *P* < 0.05 for location C). The current year increment of saplings was largest in the 25 m wide clear-cut strips in all the three locations and was significantly larger than uncut strips and control (Table 2). No significant difference was found for the current year increment between the clear-cut strip with the width of 15, 20 m, uncut strip and the control except for location A. For branch length of the first whorl, significant differences among the strip types and control were confirmed for all the three locations (F = 6.20, *P* < 0.001 for location A; F = 11.17, *P* < 0.001 for location B; F = 11.57, *P* < 0.001 for location C), and the orders follow: clear-cut strip > control > uncut strip for all the three locations. In HSD-Tukey analysis, the branch length of clear-cut strip with width of 15 and 25 m were all significantly larger than uncut strips and control for location A, and the branch length of clear-cut strip with the width of 25 m was significant larger than the width of 15 and 20 m, uncut strip and control for location B and C. No statistical difference was also found between the uncut strip and control for all the three locations. Statistically significant differences were confirmed as for the number of branches for the first whorl among strip types for location A, B, and C (F = 12.02, *P* < 0.05 for location A; F = 17.46, *P* < 0.01 for location B; F = 16.85, *P* < 0.01 for location C). The number of the branches of the first whorl from clear-cut strips were significantly larger than the uncut strip and control, and follows the trend: clear-cut strip > uncut strip > control for all the three locations. No difference was found between the uncut strip and control for all the three locations.

**Table 1:** Ages and heights of the sample saplings of *Pinus tabuliformis* from clear-cut strips, uncut strips, and control area in the western part of Liaoning Province.

Location	Strip types	Sapling density (N/ha)	Number of samples	Sapling age (year)				Sapling height (cm)		
				Mean ± Std	Min	Max		Mean ± Std	Min	Max
A	Clear-cut strip	33,450	41	19 ± 2^a^	13	24		172.6 ± 41.3^a^	79.0	288.5
	Uncut strip	24,750	24	19 ± 2^a^	12	23		155.1 ± 55.1^a^	75.0	313.0
	Control	16,750	13	18 ± 2^a^	14	20		150.6 ± 73.8^a^	10.5	276
B	Clear-cut strip	30,525	43	18 ± 2^a^	13	22		165.3 ± 40.2^a^	75.5	267.0
	Uncut strip	17,325	25	18 ± 3^a^	11	22		158.4 ± 50.6^a^	77.0	305.0
	Control	16,800	13	18 ± 2^a^	14	20		151.6 ± 72.9^a^	20.0	276.0
C	Clear-cut strip	30,425	43	17 ± 3^a^	12	21		166.0 ± 40.7^a^	75.5	257.0
	Uncut strip	17,325	25	18 ± 3^a^	11	22		204.0 ± 63.5^b^	100.0	300.0
	Control	16,800	13	18 ± 2^a^	13	20		177.6 ± 78.3^a^	100.0	300.0

**Note:**

The superscript with the same lowercase letter showed no significant difference within each location.

**Table 2:** Height and coverage for the shrubs and herbs from clear-cut strip, uncut strip and control.

Location	Strip types	Number of samples	Height of shrubs (cm)			Coverage of shrubs (%)			Height of herbs (cm)			Coverage of herbs (%)		
			Mean ± Std	Min	Max	Mean ± Std	Min	Max	Mean ± Std	Min	Max	Mean ± Std	Min	Max
A	Clear-cut strip	9	35.7 ± 16.7^a^	13.7	68.3	7.2 ± 4.8^a^	0.2	15.0	31.8 ± 11.5^a^	20.0	54.0	20.9 ± 9.6^a^	5.3	37.5
	Uncut strip	6	21.2 ± 15.0^a^	7.3	48.3	3.2 ± 3.1^a^	0.5	9.0	19.4 ± 9.7^b^	5.0	29.0	8.2 ± 3.2^b^	4.2	11.5
	Control	3	18.4 ± 14.5^a^	8.5	35.0	0.7 ± 0.5^a^	0.1	15.0	19.2 ± 5.4^b^	14.2	25.0	6.8 ± 3.2^b^	3.2	8.9
B	Clear-cut strip	9	39.6 ± 16.7^a^	10.3	59.8	6.7 ± 4.3^a^	0.1	12.8	25.4 ± 12.2^a^	7.7	45.8	12.5 ± 11.5^a^	0.6	32.0
	Uncut strip	6	23.1 ± 14.1^ab^	5.5	39.2	5.0 ± 3.5^a^	0.5	8.7	21.4 ± 7.7^a^	15.5	36.6	19.7 ± 6.4^a^	9.4	28.0
	Control	3	16.4 ± 22.3^b^	11.5	25.0	5.1 ± 3.8^a^	1.7	10.5	21.3 ± 6.7^a^	12.0	27.4	6.5 ± 1.7^a^	4.7	8.1
C	Clear-cut strip	9	40.6 ± 18.0^a^	8.0	60.0	6.3 ± 4.2^a^	0.2	12.5	37.2 ± 22.6^a^	10.9	75.0	6.9 ± 3.7^a^	1.7	12.7
	Uncut strip	6	22.1 ± 14.3^ab^	4.0	38.3	5.3 ± 4.3^a^	0.5	10.0	27.9 ± 14.1^a^	12.5	53.0	4.1 ± 2.7^a^	0.5	8.6
	Control	3	10.4 ± 4.0^b^	7.7	15.0	4.8 ± 3.7^a^	1.7	10.0	28.6 ± 17.1^a^	10.9	45.0	4.0 ± 2.5^a^	0.5	6.3

**Note:**

The superscript with the same lowercase letter showed no significant difference within each location.

### Photosynthetic rate of saplings

For the large saplings from location A, strip type was a significant effect on the net photosynthesis rates (F = 90.90, *P* < 0.05) but the time period was not (F = 1.75, *P* = 0.16). In contrast, both of strip type (F = 16.37, *P* < 0.05; F = 13.53, *P* < 0.05) and time period (F = 20.31, *P* < 0.05; F = 3.81, *P* < 0.05) have significant effect on the stomatal conductance and transpiration rates. For the large saplings from location B, both of strip type and time period have significant effect on net photosynthesis rates (F = 73.14, *P* < 0.01 for strip type, F = 7.32, *P* < 0.01 for time period), stomatal conductance (F = 16.23, *P* < 0.01 for strip type, F = 20.88, *P* < 0.01 for time period) and transpiration rates (F = 16.80, *P* < 0.01 for strip type, F = 6.01, *P* < 0.01 for time period). For the large saplings from location C, both of strip type and time period have significant effect on net photosynthesis rates (F = 73.57, *P* < 0.01 for strip type, F = 7.48, *P* < 0.01 for time period), stomatal conductance (F = 12.34, *P* < 0.01 for strip type, F = 18.68, *P* < 0.01 for time period) and transpiration rates (F = 17.17, *P* < 0.01 for strip type, F = 5.48, *P* < 0.01 for time period).

**Figure 2:**
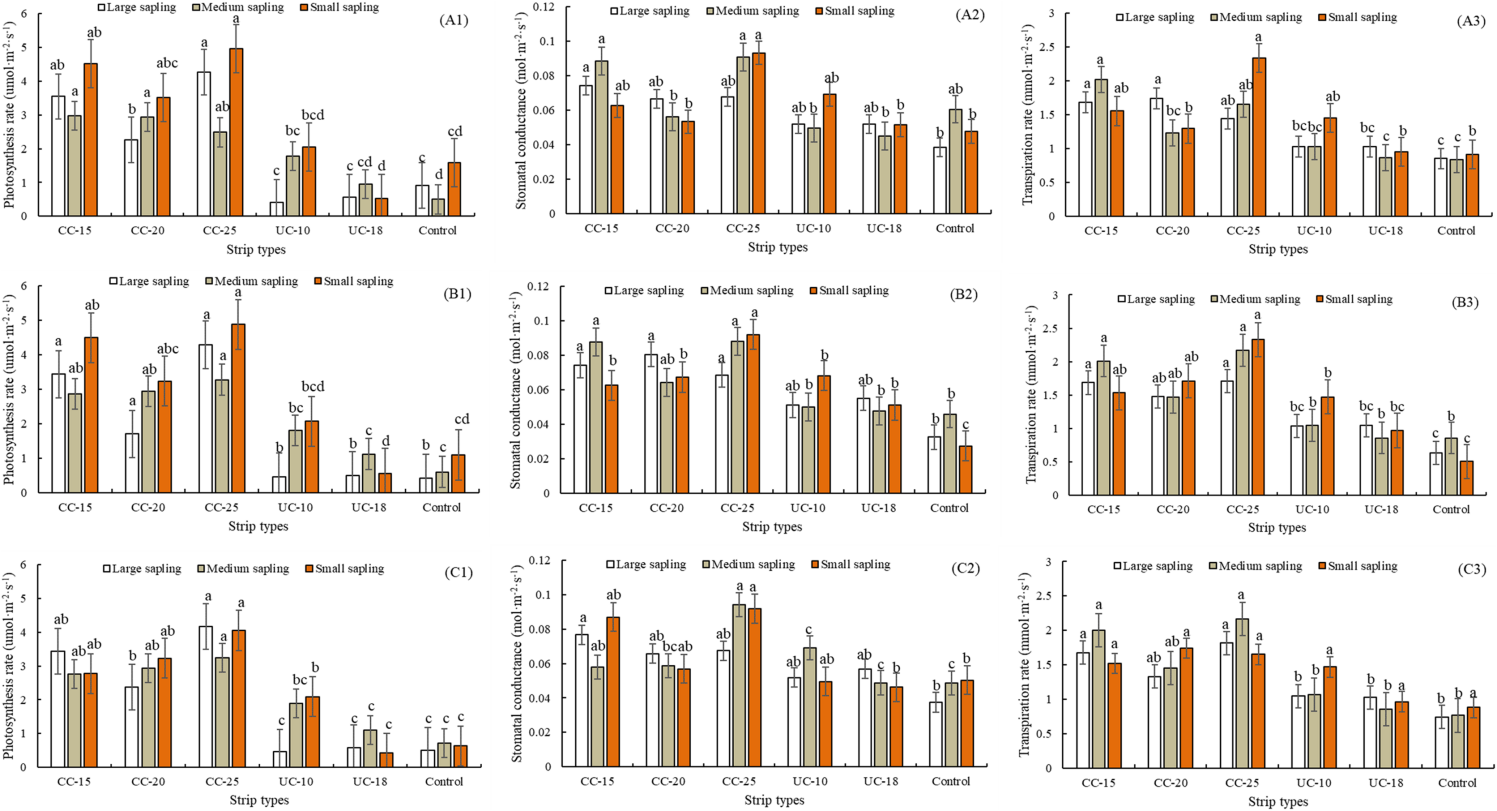
Net photosynthesis rates, stomatal conductance, and transpiration rates of large, medium, and small *Pinus tabuliformis* saplings from clear-cut strips, uncut strips, and the control. CC, clear-cut strip; UC, uncut strip; A1–A3, B1–B3, and C1–C3 indicate the photosynthesis rates, stomatal conductance, transpiration rates from location A, B, and C, respectively. The same lowercase letter above the bar for the large, medium, and small sized sapling, respectively, showed no significant difference within each location.

**Figure 3:**
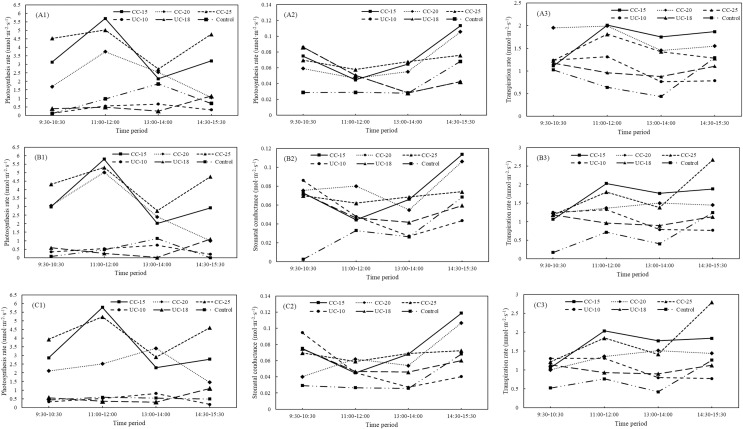
Variations in net photosynthesis rate, stomatal conductance, and transpiration rate of large *Pinus tabuliformis* sapling from clear-cut, uncut strip, and control between different periods. CC, clear-cut strip; UC, uncut strip. A1–A3, B1–B3, and C1–C3 indicate the photosynthesis rates, stomatal conductance, transpiration rates from location A, B, and C, respectively.

As for the large sapling across all the strip types from location A, the largest values of net photosynthesis rates, stomatal conductance, and transpiration rates were 4.265 umol·m^−2^·s^−1^ for the 25 m width, 0.0743 mol·m^−2^·s^−1^ for the 15 m width, and 1.738 mmol·m^−2^·s^−1^ for the 20 m widths of the clear-cut strips, respectively (Figs. 2A1–2A3). The control has the lowest stomatal conductance and transpiration rates but not the lowest net photosynthesis rates. As for the large saplings from location B and C, the net photosynthesis rates, stomatal conductance, and transpiration rates were also the largest for 25, 15, and 20 m width, respectively, and the control has the lowest stomatal conductance and transpiration rates (Figs. 2B1–2B3 and 2C1–2C3). Across four time periods (9:30–10:30, 11:00–12:00, 13:00–14:00, 14:30–15:30), net photosynthesis rates (F = 10.59, *P* < 0.05), stomatal conductance (F = 6.21, *P* < 0.05), and transpiration rates (F = 1.80, *P* < 0.05) for the large saplings from location A were significantly larger than uncut strips and control. The net photosynthesis rate was the largest in the clear-cut strips at 11:00–12:00 and that in the uncut strip with a width of 10 m and control was the largest at 13:00–14:00 (Figs. 3A1–3A3). As for the location B and C, the net photosynthesis rates of the clear-cut strip across four time periods were significantly larger than the uncut strip and control (Figs. 3B1 and 3C1). The stomatal conductance was the largest at the period of 14:30–15:30 from clear-cut strip with the width of 15 m for both of location B and C (Figs. 3B2 and 3C2), and the same to the transpiration rates (Figs. 3B3 and 3C3).

**Figure 4:**
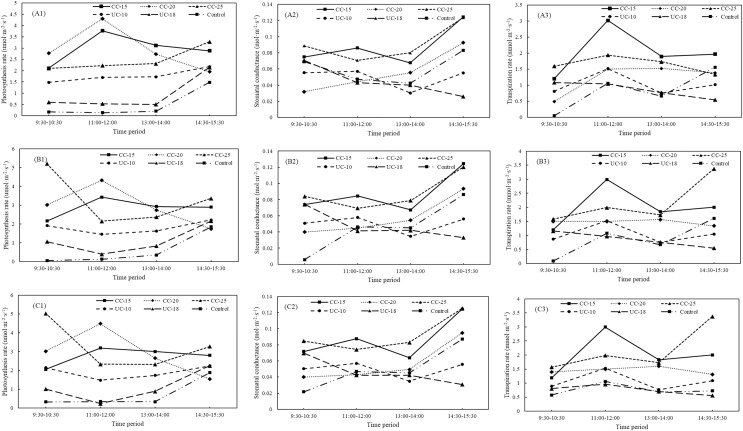
Variations in net photosynthesis rate, stomatal conductance, and transpiration rate of middle *Pinus tabuliformis* sapling from clear-cut, uncut strips, and control between different periods. CC, clear-cut strip; UC, uncut strip. A1–A3, B1–B3, and C1–C3 indicate the photosynthesis rates, stomatal conductance, transpiration rates from location A, B, and C, respectively.

For the medium-sized sapling from location A, strip type showed statistically significant effect on the photosynthesis rates (F = 25.71, *P* < 0.05), stomatal conductance (F = 24.21, *P* < 0.05), and transpiration rates (F = 25.13, *P* < 0.05). In contrast, time period was statistically significant only for the stomatal conductance (F = 16.92, *P* < 0.05), and transpiration rates (F = 10.08, *P* < 0.05). Notably, the largest photosynthesis rates was from 25 m width of the clear-cut strip with 3.307 umol·m^−2^·s^−1^, and the smallest values is from control with 0.499 umol·m^−2^·s^−1^ (Fig. 2A1). The largest stomatal conductance and transpiration rates were from the 25 and 15 m width, respectively, for the clear-cut strip, and the control was the lowest. Notably, the stomatal conductance and transpiration rates of medium-sized saplings from the clear-cut strips is the largest, followed by the uncut strips and control (Figs. 2A2–2A3). Overall, net photosynthesis rates (F = 4.63, *P* < 0.05), stomatal conductance (F = 5.05, *P* < 0.05), and transpiration rates (F = 3.02, *P* < 0.05) for the medium-sized saplings from the clear-cut strips were the largest across the four time periods, and the control was the smallest for net photosynthesis rates and transpiration rates, but not stomatal conductance (Figs. 4A1–4A3). Overall, also for location B and C, the net photosynthesis rates, transpiration rates, and stomatal conductance for the clear-cut strip were larger than the uncut strip and control (Figs. 4B1–4B3, and 4C1–4C3).

**Figure 5:**
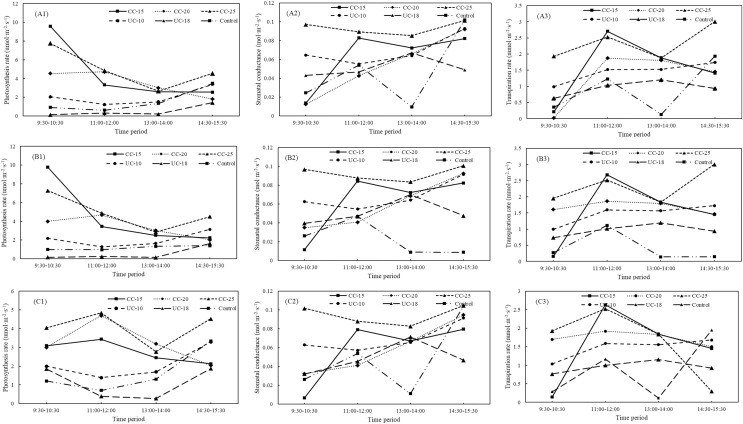
Variations for net photosynthesis rate, stomatal conductance, and transpiration rate for small *Pinus tabuliformis* saplings from clear-cut, uncut strip and control between different periods. CC, clear-cut strip; UC, uncut strip. A1–A3, B1–B3, and C1–C3 indicate the photosynthesis rates, stomatal conductance, transpiration rates from location A, B, and C, respectively.

As for the small sapling from location A, both of strip type and time period were confirmed to show statistically significant differences for net photosynthesis rates (F = 43.00, *P* < 0.05; F = 14.77, *P* < 0.05), stomatal conductance (F = 16.88, *P* < 0.05; F = 25.58, *P* < 0.05), and transpiration rates (F = 14.45, *P* < 0.05; F = 12.16, *P* < 0.05). Notably, the net photosynthesis rates for the clear-cut strip was still the largest. Different from the large and middle-sized sapling, the net photosynthesis rates for the uncut strip with 18 m was the smallest (Fig. 2A1). On the whole, the clear-cut strip has the largest stomatal conductance and transpiration rates and the control has the smallest values (Figs. 2A2–2A3). Across four time periods, net photosynthesis rates (F = 13.31, *P* < 0.05), stomatal conductance (F = 8.24, *P* < 0.05), and transpiration rates (F = 4.85, *P* < 0.05) for the small saplings were significantly larger than uncut strips and control. The net photosynthesis rates of small-sized saplings decreased from 9:30–10:30 to 13:00–14:00 and then increased in the clear-cut strips. In the uncut strips and control, the lowest values were determined at 11:00–12:00 (Fig. 5A1). The stomatal conductance increased, except for the clear-cut strip with a width of 25 m (Fig. 5A2). The maximum transpiration rate was reached at 11:00–12:00 in clear-cut strips and at 13:00–14:00 in uncut strips. The net photosynthesis rate, stomatal conductance, and transpiration rate of large, medium, and small saplings from the different types of strips exhibit the same order: clear-cut strips > uncut strips > controls, which is similar to the regularity to the location B and C (Figs. 5B1–5B3, and 5C1–5C3).

### Diversity of herb and shrub and water content of decomposition layer

Across all the strip types for the three locations, a total of 21 understory herbs and 12 shrubs were observed. Height and coverage for shrubs and herbs were showed in Table 3. The biological diversity (Shannon–Wiener index) of shrubs and herbs from clear-cut strips, uncut strips, and the control was calculated for three locations. No statistically significant effect of the strip type on the biodiversity of herbs and shrubs was observed (F = 2.16, *P* > 0.05 for herbs, F = 0.49, *P* > 0.05 for shrubs for location A; F = 2.08, *P* > 0.05 for herbs, F = 1.29, *P* > 0.05 for shrubs for location B; F = 2.44, *P* > 0.05 for herbs, F = 1.33, *P* > 0.05 for shrubs for location C). As for location A, the biological diversity of herbs in treatments with widths of 15, 20, and 25 m is 1.723, 1.180, and 0.928, respectively, and that in uncut strips with widths of 10 and 18 m and the control is 1.518 and 0.843 and 1.372, respectively. The biological diversity of shrubs in clear-cut strips with widths of 15, 20, and 25 m is 0.868, 0.898, and 0.693, respectively, and that in uncut strips with widths of 10 and 18 m and control is 0.980 and 0.915 and 0.786, respectively. The largest biological diversity of herbs with 1.655 and shrubs with 1.811 were from clear-cut strips with widths of 15 from location A and B, and 1.685 and 1.711 were also from clear-cut strips with widths of 15 from location B, respectively. Across the clear-cut strip, the control has the largest water content for both of decomposition and semi-decomposition layer, and the clear-cut strip was the smallest for decomposition and semi-decomposition layer (Table 4). However, the strip type was not a statistically significant treatment for the water content of decomposition (F = 1.15, *P* = 0.39) and semi-decomposition layer (F = 1.08, *P* = 0.42) for location A. On the whole, there was no significant difference of the water content for decomposition layer from three locations (Table 4), and the water content for the semi-decomposition layer from the clear-cut strip was significantly larger than the control and uncut strip for all the three locations (Table 4).

**Table 3:** The current year increment of sapling height (SH), branch length (BL) and number of branches (NB) of the first whorl of sample saplings from clear-cut strips, uncut strips, and control.

Location	Strip type	Strip width	Number of samples	SH (cm)	BL (cm)	NB
	Clear-cut strip	15 m	14	15.6 ± 5.9^a^	10.9 ± 4.7^ab^	3.4 ± 1.1^a^
A		20 m	15	14.8 ± 5.4^ab^	8.9 ± 5.2^bc^	3.1 ± 0.8^a^
		25 m	12	21.2 ± 13.0^c^	12.2 ± 2.6^a^	3.7 ± 0.7^a^
	Uncut strip	10 m	12	9.7 ± 3.5^b^	6.0 ± 1.0^c^	2.1 ± 0.7^b^
		18 m	12	9.7 ± 4.0^b^	6.1 ± 2.1^c^	1.7 ± 0.9^b^
	Control	–	13	10.7 ± 5.7^b^	6.3 ± 2.3^c^	1.9 ± 0.7^b^
	Clear-cut strip	15 m 20 m	15 15	15.1 ± 4.2^a^ 14.8 ± 4.9^a^	10.5 ± 4.0^a^ 10.0 ± 3.3^a^	3.1 ± 0.8^a^ 2.9 ± 0.5^a^
		25 m	13	23.7 ± 21.8^b^	13.0 ± 2.9^b^	3.8 ± 0.7^b^
B	Uncut strip	10 m	12	9.9 ± 4.0^a^	6.0 ± 1.0^c^	2.0 ± 0.8^c^
		18 m	13	9.9 ± 2.3^a^	6.2 ± 1.1^c^	2.0 ± 0.5^c^
	Control	–	13	10.1 ± 3.5^a^	6.9 ± 4.1^c^	1.8 ± 0.8^c^
C		15 m	15	15.1 ± 4.2^b^	10.6 ± 3.9^a^	3.1 ± 0.8^a^
	Clear-cut strip	20 m	15	14.8 ± 4.9^b^	9.9 ± 3.1^a^	2.9 ± 0.5^b^
		25 m	13	23.5 ± 21.8^a^	13.5 ± 3.0^b^	3.7 ± 0.7^b^
	Uncut strip	10 m	12	9.9 ± 4.0^b^	6.0 ± 1.1^c^	2.1 ± 0.7^c^
		18 m	13	13.4 ± 8.4^b^	6.7 ± 1.6^c^	2.0 ± 0.5^c^
	Control	–	13	11.3 ± 5.2^b^	6.6 ± 4.3^c^	1.8 ± 0.8^c^

**Notes:**

The values in the table were mean ± SE. SE is the standard error of the mean for each statistics.

The superscript with the same lowercase letter showed no significant difference within each location.

**Table 4:** Water content of decomposition and semi-decomposition layer from clear-cut strips with widths of 15, 20, and 25 m, uncut strips with widths of 10 and 18 m, and control.

Location	Strip types	Number of sample	Clear-cut strip			Uncut strip		Control
			15 m	20 m	25 m	10 m	18 m	
A	Decomposition (%)	18	22.1 ± 2.3^a^	24.2 ± 2.0^a^	29.8 ± 2.1^a^	24.2 ± 1.5^a^	20.4 ± 1.4^a^	31.0 ± 1.1^a^
	Semi-decomposition (%)	18	28.6 ± 1.8^a^	33.5 ± 1.6^a^	37.1 ± 1.4^a^	45.5 ± 1.6^b^	45.1 ± 1.2^b^	47.0 ± 0.9^b^
B	Decomposition (%)	18	19.7 ± 1.1^a^	23.8 ± 1.8^a^	32.4 ± 3.6^a^	26.6 ± 1.1^a^	28.6 ± 9.9^a^	33.1 ± 1.2^a^
	Semi-decomposition (%)	18	32.9 ± 4.0^a^	29.9 ± 1.4^a^	33.7 ± 2.7^a^	48.7 ± 3.7^b^	48.2 ± 3.2^b^	49.6 ± 2.1^b^
C	Decomposition (%)	18	24.6 ± 6.4^a^	27.1 ± 1.4^a^	29.8 ± 1.2^a^	25.9 ± 4.6^a^	29.9 ± 7.1^a^	33.0 ± 7.7^a^
	Semi-decomposition (%)	18	28.8 ± 3.1^a^	34.3 ± 3.2^a^	33.5 ± 2.9^a^	43.7 ± 6.6^b^	44.8 ± 2.9^b^	43.4 ± 0.8^b^

**Note:**

The superscript with the same lowercase letter showed no significant difference within each location for decomposition and semi-decomposition, respectively.

## Discussion

The growth of saplings indicates whether the saplings could survive under the mature trees or not in the future, and thus determines the forest stands dynamics (Niinemets, 2000). Our study showed that the clear-cut strip promoted the growth of the saplings regarding to the current year increment, length and number of branches for the first whorl (Table 3). The branch length of saplings in the uncut strips and control also increase, albeit at a slow rate (Noémie et al., 2011). Branch length for the first whorl significantly differed between the 20 m and 25 width for the clear-cut strip (Table 3). Light availability caused by strip widths is claimed to be the main factor in determining the branch attributes of the saplings (Finzi & Canham, 2000; Nemec, Parish & Goudie, 2012). The number of branches in the uncut strips and control is only 1.8 and 1.5, respectively, that is, significantly smaller than in the clear-cut strips (Table 3). The smaller numbers of branch for the saplings from the uncut strip and control may enlarge the risk of death of the saplings because the photosynthetic capacity also reduced (Pearcy & Yang, 2010).

Saplings from the clear-cut strip with a width of 25 m have the largest photosynthetic capacity compared with the other strip widths and control. However, a larger clear-cut strip is impractical for seed transmission, because seed production is the main process to ensure the successful regeneration (Kozlowski, 2002; Woziwoda et al., 2019). The stomatal conductance and transpiration rate are also useful indicators of the potential photosynthetic capacity of saplings (Egea, Verhoef & Vidale, 2011; Houshmandfar et al., 2021). Based on the present study, the stomatal conductance of large, medium, and small saplings in clear-cut strips is the largest and that determined for the control is the smallest. The transpiration rates of large, medium, and small saplings from clear-cut strips are the largest and those obtained for the control are the smallest. The results of our study indicated that the clear-cut strip increased the sapling vitality (Vitali et al., 2016). However, the clear-cut strip width should be considered in practice because the wider width would lead to the impractical process for seed transmission. In the next step, the response of forest trees to edge created by the clear-cut strip with different widths should be further investigated.

Conservation of biological diversity of herbs and shrubs in the forests is essentially important in the sustainable forest management (Moore & Allen, 1999). The present study showed the biodiversity of herbs and shrubs is the largest in uncut strips, followed by clear-cut strips with a width of 15 and 20 m and the control. The clear-cut strip with a width of 25 m exhibits the smallest biodiversity in our study. The results of our study also claimed that the more light interception in the wider clear-cut strip tends to change the crown more flatter (Juchheim et al., 2017). The reason may be the more flatterer crown and high density of the saplings has reduced the biodiversity of the herbs and shrubs (Sampo & Pauline, 2001; Pauline, Kaufmann & Ryan, 2005). This might be due to the significant improvement of the sapling growth in clear-cut strips compared with uncut strips and the control due to the increase in light interception. The high density of saplings in clear-cut strips hinders the regeneration of shrubs and herbs in the understory. Therefore, strip clear-cutting increases the sapling height and branch length but decreases the understory shrub and herb regeneration unless other sapling management practices are applied.

The regeneration was affected not only by the strip widths, but also the relative position of strips, because the microclimate condition will be affected by the widths of the neighboring strips (Klenner & Sullivan, 2003). In our study, the regeneration from the 18 m of the uncut strip was similar to the control, because the 18 m strip is located in the immediate vicinity of control stand and the microclimate was similar. Therefore, to ensure the comparability for the three locations, the relative positions for the clear-cut strips, uncut strips and control were the same for all the three locations. With the sapling growing, the competition between saplings would significantly increase and the growth of seedlings would be affected (Noémie et al., 2011). Thus, the sustainability for the natural regeneration would be impeded. In addition, the relationship between the sapling growth and the growth of the individual tree nearest to the strip edge should be also taken into consideration.

## Conclusions

In the mature *Pinus tabuliformis* plantations from the three locations in western part of the Liaoning Province in northeast China, strip clear-cutting significantly affected the sapling growth, diversity of shrubs and herbs, but not the water content of decomposition and semi-decomposition layer. The following trends were observed for the current year increment of sapling height, branch length of the first whorl: clear-cut strips > control > uncut strips. The number of branches for the first whorl can be ordered as follows: clear-cut strips > uncut strips > control. The saplings from the clear-cut strip with a width of 25 m have the largest photosynthetic. The transpiration rates of large, medium, and small saplings in the clear-cut strip are the largest and those obtained for the control are the smallest. The water content of the decomposition and semi-decomposition layer in the control is the largest compared to clear-cut and uncut strips and the treatment was not statistically significant. Widths of clear-cutting strip significantly affected the length and number of the branches for the first whorl. Photosynthetic capacity of the saplings expressed statistically significant between strip types, and the time periods only showed significant effect for stomatal conductance and transpiration rates. With the increasing of sapling size, the reserve density for saplings should be taken into consideration to favour the growth of seedlings and saplings.

## Supplemental Information

10.7717/peerj.13341/supp-1 Supplemental Information 1 Dateset for location 1.

10.7717/peerj.13341/supp-2 Supplemental Information 2 Dateset for location 2.

10.7717/peerj.13341/supp-3 Supplemental Information 3 Dateset for location 3.

## Additional Information and Declarations

### Data Availability

The following information was supplied regarding data availability: The raw data is available in the Supplemental File.
